# Relationship between alcohol co-ingestion and outcome in profenofos self-poisoning – A prospective case series

**DOI:** 10.1371/journal.pone.0200133

**Published:** 2018-07-05

**Authors:** H. K. Jeevan Dhanarisi, Indika B. Gawarammana, Fahim Mohamed, Michael Eddleston

**Affiliations:** 1 South Asian Clinical Toxicology Research Collaboration, Faculty of Medicine, University of Peradeniya, Peradeniya, Sri Lanka; 2 Department of Medicine, Faculty of Medicine, University of Peradeniya, Peradeniya, Sri Lanka; 3 Department of Pharmacology, School of Medical Sciences, Sydney, Australia; 4 Department of Pharmacy, Faculty of Allied Helath Sciences, University of Peradeniya, Peradeniya, Sri Lanka; 5 Pharmacology, Toxicology, & Therapeutics, University/BHF Centre for Cardiovascular Science, University of Edinburgh, United Kingdom; National Yang-Ming University, TAIWAN

## Abstract

**Introduction:**

The importance of alcohol co-ingestion for outcome in organophosphorus (OP) insecticide self-poisoning has only been studied for the relatively hydrophilic dimethyl insecticide, dimethoate. We aimed to assess the effect of alcohol in acute poisoning with the lipophilic S-alkyl OP insecticide, profenofos.

**Methodology:**

Demographic and clinical data, including an alcohol history, were prospectively collected from all cases of acute poisoning with agricultural profenofos EC50 presenting to two Sri Lankan hospitals over seven years.

**Results:**

Of 1859 patients with acute OP insecticide self-poisoning, 243 (13.1%) reported ingestion of profenofos (male 182/243, 74.9%). Alcohol co-ingestion was reported by 64/243 (26.3%). All patients reporting alcohol co-ingestion were male (64/64 [100%] vs 118/179 [65.9%] not reporting alcohol ingestion, p<0.001). More patients reporting alcohol co-ingestion died (10/64 [15.6%] vs 10/179 [5.6%]; p = 0.013) and required intubation (13/64 [20.3%] vs 16/179 [8.9%], p = 0.016) compared to those who did not co-ingest alcohol. Using multi-logistic regression, controlling for the estimated dose ingested, age (OR 11.1 [2.5 to 48.9] for age > 35 years vs ≤35 years) and alcohol co-ingestion (OR 3.1 [1.2 to 7.9]) were independently associated with increased risk of death. Increased risk of intubation was independently associated with age (OR 3.2 [1.6 to 6.6] for age > 35 years vs ≤35 years) and alcohol co-ingestion (OR 3.2 [1.6 to 6.4]).

**Conclusion:**

A history of alcohol co-ingestion, as well as older age, is independently associated with worse outcome in patients’ self-poisoned with profenofos.

## Introduction

Acute pesticide self-poisoning is a major public health problem in many developing countries, killing tens of thousands of people each year [1]. The World Health Organization (WHO) recognizes pesticide poisoning to be one of the three most important means of suicide worldwide [2].

Organophosphorus (OP) insecticides have been responsible for many of these deaths in the developing world and in Sri Lanka for the last 40 years [3, 4]. The problem is particularly severe in rural Asian communities where organophosphate insecticides are widely available for use in agriculture and therefore accessible for self-harm at times of stress. District hospitals bear the brunt of the problem, many seeing hundreds of OP insecticide poisoned patients each year [3] with 30–50% requiring ventilatory support for up to several weeks [5, 6].

OP insecticide toxicity results from inhibition of acetylcholinesterase (AChE), causing accumulation of acetylcholine, and overstimulation at cholinergic synapses throughout the body. This results in an ‘acute cholinergic crisis’ with bradycardia, hypotension, coma, and acute respiratory failure [7]. Respiratory failure may also be delayed, occurring after several days due to neuromuscular junction dysfunction and termed the intermediate syndrome [8]. Butyrylcholinesterase (BuChE) is also inhibited by OP insecticides and is used as a marker of exposure [9].

After multiple pesticide bans over the last two decades [10], the S-alkyl OP insecticide profenofos, formulated as a 50% emulsifiable concentrate (EC50), is now one of the most common OP insecticides ingested for self-harm in Sri Lanka. Profenofos is classified as a WHO Class II moderately toxic OP insecticide (rat oral LD50 358 mg/kg) with a case fatality of around 11% [11]. It is also highly lipophilic, with slow elimination from the body due to distribution to body fat stores [7]. This lipophilicity can result in recrudescence of toxicity and clinical symptoms after several days when patients are apparently getting better. This is quite different for example to the relatively hydrophilic OP insecticide, dimethoate, that was the most important cause of death in the 2000s [12].

Ethanol is an important risk factor for pesticide poisoning and is commonly also co-ingested during self-poisoning with pesticides. Clinical experience suggests that alcohol co-ingestion makes management more difficult [13, 14]. In a study of self-poisoning with dimethoate EC40, alcohol intoxication was associated with ingestion of larger amounts of pesticide and worse outcome [15]. It is not yet clear whether this is true for other OP insecticides, especially lipophilic compounds. In this study, we have compared the clinical outcomes of acute self-poisoning with profenofos EC50, with or without a history of alcohol co-ingestion.

## Materials and methods

This was a prospective case series of acute profenofos EC50 insecticide self-poisoning presented between 01 January 2010 to 31 March 2017 to the specialized Toxicology Unit, Teaching Hospital Peradeniya, and to Teaching Hospital Kurunegala, Sri Lanka. Informed written consent was obtained from all patients or their relatives; the study was approved by Human Research and Ethics Committee of the Faculty of Medicine, University of Peradeniya.

Demographic and clinical data, along with a history of alcohol co-ingestion, were collected using a structured questionnaire by medically trained researchers. Ingestion of profenofos with or without alcohol was ascertained by questioning the patient and family, as well as medical notes from transferring hospitals and bottles brought with the patient (this has previously been found to be a good way of identifying the pesticide ingested, when compared to laboratory analyses of blood samples) [12, 16]. The volume of ingestion was estimated from the patient’s recall or by the amount of pesticide remaining in the bottle brought to hospital when available.

Erythrocyte cholinesterase activity was determined using Test-mate ChE Cholinesterase Test System (model 400) (EQM Research, Inc., Cincinnati, OH) (DOI: https://dx.doi.org/10.17504/protocols.io.nn2ddge) using blood sample collected by trained clinical research assistants on admission as per our previous practice [9, 17]. This was not done for all patients but for those in “Development of biomarkers of neuromuscular junction dysfunction and neurocognitive dysfunction after toxic injuries” study. This equipment consists of a photometric analyzer with a microprocessor and a temperature sensor that compensates for ambient temperature. This work has shown good correlation between the Test-mate AChE assay results with the standard laboratory assay, with a Spearman's correlation coefficient of 0.87 (95% CI 0.81 to 0.91) [17]. The test system is based on the Ellman colorimetric method in which acetylthiocholine is hydrolyzed by AChE, producing carboxylic acid and thiocholine, which reacts with the Ellman reagent (dithionitrobenzoic acid) and turns yellow. The rate of color formation is proportional to the amount of AChE [18, 19].

Data analysis was performed using Stata v14 statistical software (StataCorp, TX) and GraphPad Prism v5 software (GraphPad, CA). Clinical characteristics were summarized using counts (percentages) for categorical variables and the median [interquartile range (IQR)] for non-normally distributed continuous variables. The t test and odds ratios (ORs) were used to compare categorical values. Multivariable logistic regression models were used to investigate whether there was an association between history of alcohol consumption, intubation and mortality.

## Results

A total of 1859 patients with acute OP insecticide poisoning presented over seven years to the two study hospitals. From this cohort, 243 (13.1%) patients reported ingesting profenofos [median age 35 (IQR 25–48)] of whom a quarter reported co-ingesting alcohol (n = 64/243, 26.3%). The majority of patients reporting alcohol co-ingestion were chronic users, consuming alcohol daily (54.7%). Patient demographics are presented in Table 1. The majority of patients were living in rural areas (84.9%). Most common reported co-morbidities which associated with both groups were respiratory diseases, heart diseases, hypertension, diabetic mellitus and psychiatric diseases.

**Table 1:** Characteristics of profenofos self-poisoned patients with or without a history of alcohol co-ingestion.

Characteristics	No alcohol n = 179	With alcohol n = 64	P- value
**Men (n [%])**	118 [65.9%]	64 [100%]	p<0.0001
**Age (yrs, median [IQR])**	32 [23 to 45]	48 [36 to 60]	p<0.0001
**Rural residence**	152 [84.9%]	56 [87.5%]	p = 0.6113
**Urban residence**	27 [15.1%]	8 [12.5%]	p = 0.6113
**Alcohol dependent (daily consumption)**	0	35 [54.7%]	NA
**Non-dependent alcohol consumption(Once or twice a week)(Once or twice a month)**	0 0 0	29 [45.3%] 18 [28.1%] 11 [17.2%]	NA NA NA
**Co-morbidities (respiratory diseases, heart diseases, hypertension, diabetic mellitus, psychiatric diseases, etc.)**	32 [17.9%]	11[17.2%]	p = 0.8898
**Ingested profenofos dose (mL, median [IQR]) ^a^(n = 147 [106 no alcohol, 41 with alcohol])**	40 [15 to 100]	30 [15 to 100]	p = 0.77
**Red cell AChE activity (mU/μmol Hb, median [IQR]) ^a^** **[n = 94 (64 no alcohol, 30 with alcohol)]**	0 [0 to 293.3]	0 [0 to 0.8]	p = 0.0559

^a^Information on estimated ingested profenofos and red cell AChE, acetylcholinesterase activity dose was available for 147 (60.5%) and 94 (38.7%) patients, respectively.

**Fig 1:**
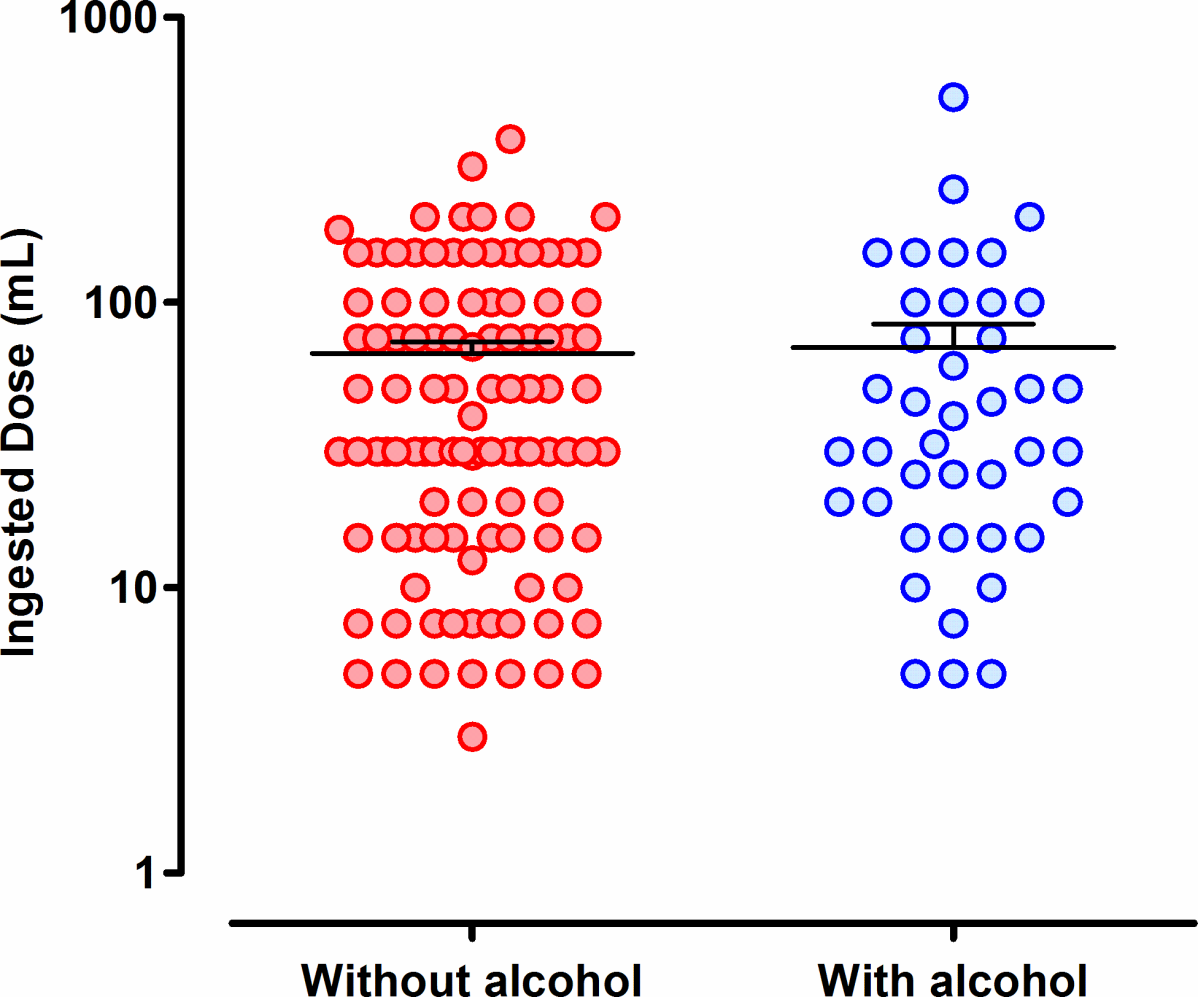
Estimated ingested profenofos dose in the two patient groups, with and without a history of alcohol co-ingestion.

**Fig 2:**
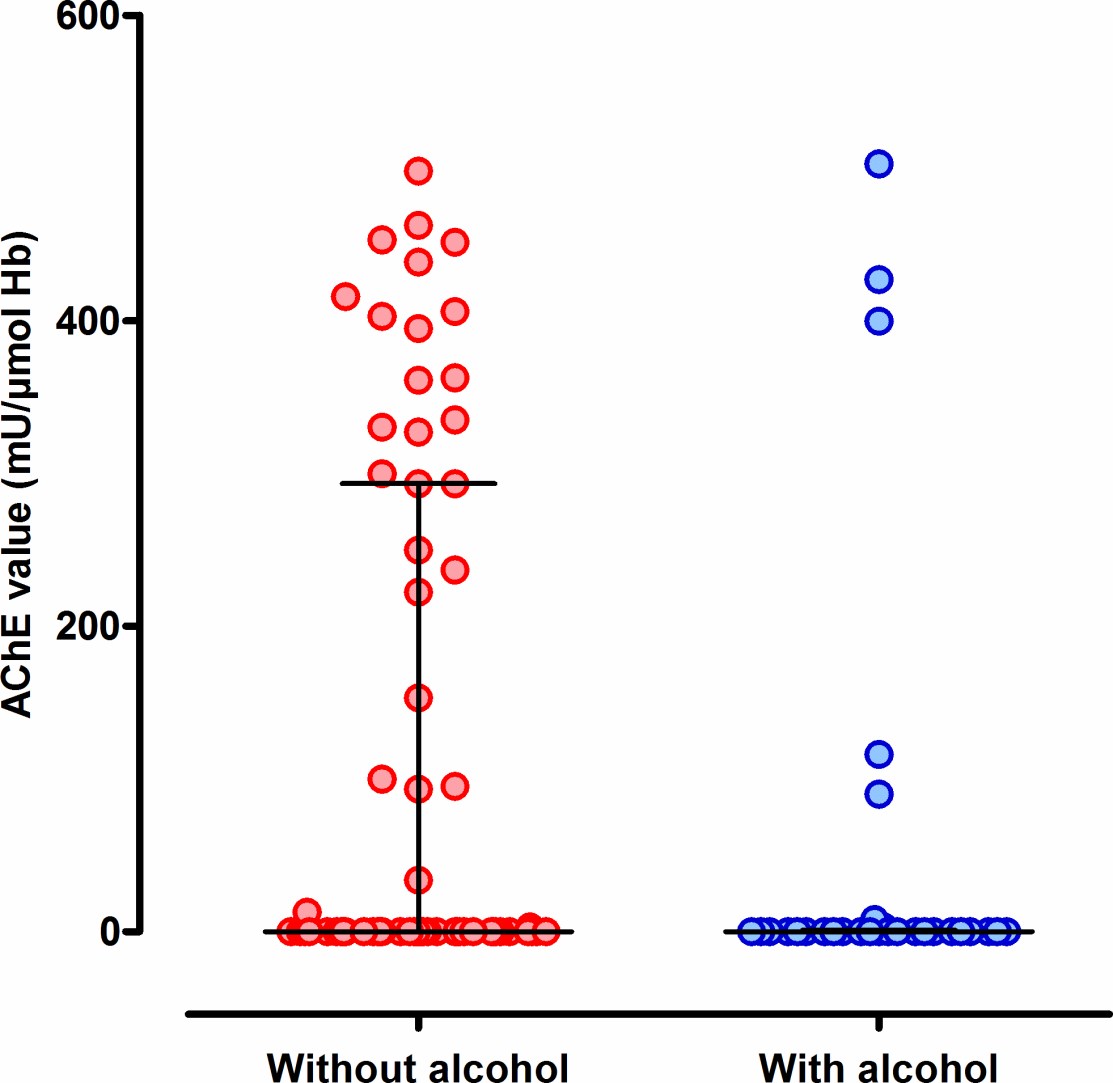
Red cell AChE values taken on admission in the two profenofos poisoned patient groups, with and without a history of alcohol co-ingestion. Data were available for 94 (64 no alcohol, 30 with alcohol) patients.

Men made up the majority of profenofos poisoning cases (182/243, 74.9%) and all of those reporting alcohol co-ingestion (64/64 [100%] vs 118/179 [65.9%] of people who did not report ingestion of alcohol, p<0.001, Table 1). Cases who co-ingested alcohol were older (Table 1). The two groups did not differ according to the reported ingested dose (Fig 1) and co-morbidities (Table 1); there was a trend towards reduced AChE activity on admission in patients co-ingesting alcohol but this did not reach significance (Table 1, Fig 2).

Patients reporting alcohol co-ingestion had a higher risk of death than those not co-ingesting alcohol (10/64 [15.6%] vs 10/179 [5.6%]; p = 0.013). They also required intubation more often (13/64 [20.3%] vs 16/179 [8.9%]; p = 0.016) and had a non-significantly and modestly longer hospital stay (median 3.6 [IQR 2.0 to 7.8] vs 3.1 [1.9 to 5.0) days; p = 0.112) than those who did not co-ingest alcohol.

Multi-logistic regression using the variables age, sex, estimated ingested profenofos dose, co-morbidities and alcohol co-ingestion showed an increased risk of death for older people (odds ratio [OR] 11.1 [2.5 to 48.9] for age greater than 35 years vs. 35 years or less) and for alcohol co-ingestion (OR 3.1 [1.2 to 7.9]). There was also an increased risk of intubation for older people (odds ratio [OR] 3.2 [1.6 to 6.6] for age greater than 35 years vs. 35 years or less) and alcohol co-ingestion (OR 3.2 [1.6 to 6.4]). The risk of death in men, controlled for age, profenofos dose, and alcohol co-ingestion, was non-significantly increased (OR 7.0 [0.9 to 53.3] while the risk of intubation was increased OR 9.8 [2.3 to 42.0]). The estimated ingested profenofos dose was not independently associated with increased risk of death (OR 1.0 [1.0 to 1.0]) or intubation (OR 1.0 [0.9 to 1.0]). And also co-morbidities were not independently associated with increased risk of death (OR 1.0 [0.3 to 3.7]).

## Discussion

Alcohol co-ingestion, as well as older age, is independently associated with worse hospital outcome in patients self-poisoned with the lipophilic S-alkyl OP insecticide profenofos. These results support the hypothesis that alcohol co-ingestion worsens outcome in OP self-poisoning and add to the previous work on dimethoate poisoning [15].

This effect may be due to larger ingestions of pesticide by intoxicated patients compared to sober patients, as previously noted for dimethoate [15], or due to an interaction of profenofos with ethanol. However, surprisingly, we found no relationship between clinical outcome and estimated ingested profenofos dose. The reason for the lack of effect of the dose in clinical outcome is unclear. Perhaps it is due to the difficulty of accurately estimating the dose ingested. However, large prospective case series of other pesticides have usually found a dose effect [20, 21]. It is possible that patient differences in profenofos metabolism [22], increasing sensitivity in some patients, might explain a difference if the lack of effect of dose can be confirmed in future studies.

There was a trend towards reduced AChE activity on presentation in patients co-ingesting ethanol, suggesting perhaps a more severe poisoning. However, profenofos is such a potent inhibitor of AChE that the enzyme is often completely inhibited in quite mild poisoning [11]. The median AChE activity was the same (0 mU/**μ**mol Hb) in both groups; there were more patients in the non-alcohol ingestion group with high AChE activity. We only sampled 35.8% and 46.9% of patients in the no-alcohol and alcohol groups, respectively, suggesting that the difference may be random. Future studies will need to analyze AChE and probably butyrylcholinesterase (BuChE) in all recruited patients as well as measure profenofos concentrations to get a better idea of the ingested dose (since AChE activity is a poor marker of dose in profenofos poisoning). A recent retrospective study of 135 OP insecticide poisoned patients admitted to five South Korean hospitals reported that blood ethanol concentration correlated with survival (although data on dose and proportion of patients who had ingested alcohol data were not presented). Using receiver operating characteristic (ROC) analysis this study identified a blood alcohol concentration of 173 mg/dL that was independently associated with death (OR 4.9 [1.5 to 16.7]) [23]. This finding differs from the dimethoate study, in which controlling in the analysis for the dimethoate concentration removed any association with ethanol, indicating that the effect of ethanol was due to higher doses of ingested OP and not due to the ethanol itself. The Korean study did not measure OP blood concentrations and so the authors were not able to test the relative importance of OP and ethanol in affecting outcome. In addition, the patients had ingested one of a variety of pesticides rather than one particular OP insecticide, increasing variability, and the data were retrospective.

Although this is the largest case series of profenofos poisoning reported to date, this study is limited by the lack of laboratory proof of exposure, its relatively small sample size, and uncertain accuracy of the reported volume ingested (due to it been based on patient recall/ volume remaining in the bottle). However, our previous work has shown that the history is highly accurate for identifying the pesticide involved in an exposure to particular pesticides [12, 16]. Measurement of plasma profenofos and blood alcohol concentrations in our cohort would have helped elucidate the relative contributions of the pesticide and alcohol to outcome and test the hypothesis that dose of profenofos is not associated with outcome. Medical records covering previous admissions or clinic appointments do not exist in the study hospitals, meaning that it was not possible to report alcohol history with that previously reported.

The present study suggests that public health campaigns to reduce alcohol consumption and increase awareness of its negative effects on health may ultimately improve outcome from profenofos (and other forms of) self-poisoning. Additional studies are required in which plasma profenofos and blood alcohol concentrations as well as AChE and BuChE activities are measured on admission in all patients to confirm exposure. These studies are important to understand the influence of co-ingested alcohol on OP severity and this may guide future integrated policy changes to improve the managementof OP poisoning and to reduce overall mortality.

## Conclusion

Reported alcohol co-ingestion is independently associated with worse hospital outcome in patients self-poisoned with the lipophilic OP insecticide profenofos. Further studies are now needed to determine the relationship between blood alcohol concentration, profenofos dose, and outcome in profenofos OP self-poisoning. Efforts to reduce deaths from profenofos self-poisoning may benefit from public health efforts focusing on reduce alcohol consumption.

## Supporting information

S1 File Structured questionnaire. (PDF)

## References

[1] Mew EJ, Padmanathan P, Konradsen F, Eddleston M, Chang SS, Phillips MR, et al The global burden of fatal self-poisoning with pesticides 2006–15: Systematic review. J Affect Disord. 2017;219:93–104. Epub 2017/05/24. doi: 10.1016/j.jad.2017.05.002 . 28535450 10.1016/j.jad.2017.05.002

[2] World Health Organization. Preventing suicide. A global imperative Geneva: WHO; 2014 2014.

[3] Eddleston M. Patterns and problems of deliberate self-poisoning in the developing world. Q J Med. 2000;93:715–31. 10.1093/qjmed/93.11.715 11077028

[4] Knipe DW, Metcalfe C, Fernando R, Pearson M, Konradsen F, Eddleston M, et al Suicide in Sri Lanka 1975–2012: age, period and cohort analysis of police and hospital data. BMC Public Health. 2014;14:839 doi: 10.1186/1471-2458-14-839 25118074 10.1186/1471-2458-14-839 PMC4148962

[5] Eddleston M, Sudarshan K, Senthilkumaran M, Reginald K, Karalliedde L, Senarathna L, et al Patterns of hospital transfer for self-poisoned patients in rural Sri Lanka: implications for estimating the incidence of self-poisoning in the developing world. Bulletin of the World Health Organization. 2006;84(4):276–82. 16628300 10.2471/blt.05.025379 PMC1950595

[6] Eddleston M, Mohamed F, Davies JO, Eyer P, Worek F, Sheriff MR, et al Respiratory failure in acute organophosphorus pesticide self-poisoning. Journal of the Association of Physicians. 2006;99(8):513–22. 10.1093/qjmed/hcl065 PMC1525210 16861715

[7] Eddleston M, Buckley NA, Eyer P, Dawson AH. Management of acute organophosphorus pesticide poisoning. The Lancet. 2008;371(9612):597–607. 10.1016/S0140-6736(07)61202-1 PMC2493390 17706760

[8] Karalliedde L, Baker D, Marrs TC. Organophosphate-Induced Intermediate Syndrome. Toxicological reviews. 2006;25(1):1–14. 16856766 10.2165/00139709-200625010-00001

[9] Worek F, Mast U, Kiderlen D, Diepold C, Eyer P. Improved determination of acetylcholinesterase activity in human whole blood. Clin Chim Acta. 1999;288(1–2):73–90. 10529460 10.1016/s0009-8981(99)00144-8

[10] Knipe DW, Gunnell D, Eddleston M. Preventing deaths from pesticide self-poisoning—learning from Sri Lanka's success. The Lancet Global Health. 2017;5(7):e651–e2. doi: 10.1016/S2214-109X(17)30208-5 28619217 10.1016/S2214-109X(17)30208-5

[11] Eddleston M, Worek F, Eyer P, Thiermann H, von Meyer L, Jeganathan K, et al Poisoning with the S-Alkyl organophosphorus insecticides profenofos and prothiofos. QJM. 2009;102:785–92. doi: 10.1093/qjmed/hcp119 19737786 10.1093/qjmed/hcp119 PMC2766103

[12] Eddleston M, Eyer P, Worek F, Mohamed F, Senarathna L, von Meyer L, et al Differences between organophosphorus insecticides in human self-poisoning: a prospective cohort study. The Lancet. 2005;366(9495):1452–9. 10.1016/S0140-6736(05)67598-8 16243090

[13] van der Hoek W, Konradsen F. Risk factors for acute pesticide poisoning in Sri Lanka. Trop Med Int Health. 2005;10:589–96. doi: 10.1111/j.1365-3156.2005.01416.x 15941423 10.1111/j.1365-3156.2005.01416.x

[14] Eddleston M, Buckley NA, Gunnell D, Dawson AH, Konradsen F. Identification of strategies to prevent death after pesticide self-poisoning using a Haddon matrix. Inj Prev. 2006;12:333–7. doi: 10.1136/ip.2006.012641 17018677 10.1136/ip.2006.012641 PMC1950775

[15] Eddleston M, Gunnell D, von ML, Eyer P. Relationship between blood alcohol concentration on admission and outcome in dimethoate organophosphorus self-poisoning. Br J Clin Pharmacol. 2009;68(6):916–9. doi: 10.1111/j.1365-2125.2009.03533.x 20002086 10.1111/j.1365-2125.2009.03533.x PMC2805864

[16] Roberts DM, Seneviratne R, Mohammed F, Patel R, Senarathna L, Hittarage A, et al Intentional self-poisoning with the chlorophenoxy herbicide 4-chloro-2-methylphenoxyacetic acid (MCPA). Annals of emergency medicine. 2005;46(3):275–84. doi: 10.1016/j.annemergmed.2005.03.016 16126140 10.1016/j.annemergmed.2005.03.016 PMC1475925

[17] Rajapakse BN, Thiermann H, Eyer P, Worek F, Bowe SJ, Dawson AH, et al Evaluation of the Test-mate ChE (cholinesterase) field kit in acute organophosphorus poisoning. Ann Emerg Med. 2011;58(6):559–64. e6. doi: 10.1016/j.annemergmed.2011.07.014 22098995 10.1016/j.annemergmed.2011.07.014

[18] von Osten JR, Tinoco-Ojanguren R, Soares AM, Guilhermino L. Effect of pesticide exposure on acetylcholinesterase activity in subsistence farmers from Campeche, Mexico. Archives of Environmental Health: An International Journal. 2004;59(8):418–25. 10.3200/AEOH.59.8.418-425 16268118

[19] Ellman GL, Courtney KD, Andres V Jr, Featherstone RM. A new and rapid colorimetric determination of acetylcholinesterase activity. Biochemical pharmacology. 1961;7(2):88–95. 13726518 10.1016/0006-2952(61)90145-9

[20] Wilks MF, Fernando R, Ariyananda P, Eddleston M, Berry DJ, Tomenson JA, et al Improvement in survival after paraquat ingestion following introduction of a new formulation in Sri Lanka. PLoS medicine. 2008;5(2):e49 doi: 10.1371/journal.pmed.0050049 18303942 10.1371/journal.pmed.0050049 PMC2253611

[21] Moon JM, Chun BJ. Acute endosulfan poisoning: a retrospective study. Human & experimental toxicology. 2009;28(5):309–16. 19755461 10.1177/0960327109106488

[22] Wing KD, Glickman AH, Casida JE. Oxidative bioactivation of S-alkyl phosphorothiolate pesticides: stereospecificity of profenofos insecticide activation. Science. 1983;219(4580):63–5. 6849116 10.1126/science.6849116

[23] Lee YH, Oh YT, Lee WW, Ahn HC, Sohn YD, Ahn JY, et al The association of alcohol consumption with patient survival after organophosphate poisoning: a multicenter retrospective study. Intern Emerg Med. 2016 Epub 2016/06/14. doi: 10.1007/s11739-016-1484-9 . 27294348 10.1007/s11739-016-1484-9

