# Supplementary material for: Relationship between alcohol co-ingestion and outcome in profenofos self-poisoning – A prospective case series
Source: PLoS One. 2018 Jul 5;13(7):e0200133. doi: 10.1371/journal.pone.0200133 (PMC6033444; doi:10.1371/journal.pone.0200133)
Supplement: S1 File — (PDF) [file pone.0200133.s001.pdf]

Treatment Detail Form Cohort/Referral Hospital

Study ID:		BHT:		Hospital Number:		Ward:	

Patient Details

First name:		Age:		Gender	
Family name:		Resident Address:			

Town/Village:		National ID:		Date of Birth	
Religion	Buddhist/Hindu/Muslim/Christian/Other	Ethnic Group:	Sinhalese/Sri Lankan Tamil / Indian Tamil/ Sri Lanka Moor/Sri Lankan Malay/Burger/Other		
Pregnant:	Yes/No	Co-morbid illness:			

Prior to Hospitalization

Ingested Poison:		Amount ingested:		Ingestion Date/Time:			
Co- ingestion with Alcohol:	Yes/No	Amount:		Brand:			
Chronic alcohol User:	Yes /No	How long:		Occasional alcohol user:	Yes/no	How often:	
Spontaneous Vomiting :	Yes/No	How Long after Ingestion:					

Details from Transferred/Peripheral Hospital

a)

Name of the transferred hospital:			
Admission date & Time:		Transferred Date& Time :	
Transferred in:	Ambulance/Private Vehicle/Hired Vehicle	Transferred with	doctor/Nurse/attendant/Other
Vomiting on admission to Transferred/peripheral Hospital:	Yes/No	Method of GI Decontamination	No GID/ Forced emesis/ Gastric Lavage / Gastric Lavage with NG Tube/ Gastric Lavage with Orogastric tube
Activated Charcoal:	given/not given	GID details from:	Patient/ Relative / Bystander/ Other

a) Antidotes and other treatment at transferred/Peripheral hospital

Atropine given	Yes/No	Dose:		Methionine	Yes/No	Pralidoxime	Yes/No	Dose:	
IV line inserted	Yes/No	Intubated	Yes/No	Intubated Date & time:		Other Treatments (list):			

Data from Referral Hospital

a)

Type of admission	Direct / Transferred	Vomiting on admission	Yes / No
Arrival to Referral hospital Date/ Time		Symptoms On admission	Symptomatic /Asymptomatic /unknown
GCS on Admission to Referral Hospital:			

b) Events Prior to Examination

Respiratory arrest	Yes / No	Cardiac arrest	Yes / No	Intubated	Yes/ No	Intubated GCS:			
Intubated Date & Time:		Seizures	Yes / No	Epileptics	Yes/ No		Dysrhythmias	Yes/No	
Hypotension	Yes / No	Pulmonary edema	Yes/No	Coma	Yes/No	Delirium	Yes/ No		
Other		Method of GI Decontamination in Referral Hospital	No GID/ Forced emesis/ Gastric Lavage/ Gastric Lavage with NG Tube/ Gastric Lavage with Orogastric tube						
Activated Charcoal	single dose/ multiple dose/ not given								

c) Antidote Treatment at Referral hospital (on admission)

Pralidoxime Given	Yes/No	Dose		Methionine/NAC Given	Yes/No	Dose	
Atropine Given	Yes/No	Dose		Other		Dose	
Data Collected by		Date and time					

Patient clinical Monitoring Sheets

Study ID:	

Time/Date	Pulse Rate	Pulse Rhythm Regular/Irregular	RR	Lungs signs (rhonchi, crept etc.	Pupil Size (mm)	Dry axillae Y/N	BP (mm Hg)	Bowel sounds (A/D/N/I)	(GCS)	Saturation	Neurological signs(ptosis, ataxia etc	Remarks (vomiting/ diarrhea/ abdominal Pain/fever/others	Treatment during Hospitalization (Atropine, PAM, Methionine/NAC others.)

Please fill the additional continuation clinical monitoring sheet if above space is not sufficient

Intubated Date & Time	Extubated Date & Time

Date & Time of On Admission Blood Collection	

Patient Status (Outcome):	Discharged Alive/ Death/Transferred/ LAMA/Missing/Retransferred
Date & Time of Status:	
